# Association of Socioeconomic Risk Factors of Food Insecurity With Post-abdominal Surgery Complications in the USA

**DOI:** 10.7759/cureus.114846

**Published:** 2026-08-20

**Authors:** Rita K Bliesner, Dominick DeMasi, Laura Cossette, Rahul Garg

**Affiliations:** 1 Osteopathic Medicine, Alabama College of Osteopathic Medicine, Dothan, USA; 2 Medicine, Alabama College of Osteopathic Medicine, Dothan, USA; 3 Research, Alabama College of Osteopathic Medicine, Dothan, USA

**Keywords:** food insecurity, gastrointestinal surgery, national inpatient sample (nis) and the healthcare cost and utilization project (hcup), postoperative complications, socioeconomic factors

## Abstract

Background

Postoperative complications are common for gastrointestinal surgeries, ranging from mild symptoms such as nausea and scarring to fatal outcomes such as hemorrhage and sepsis. Comorbidities and overall preoperative health of a patient impact both surgical outcomes and postoperative complications. Socioeconomic risk factors such as food deserts, healthcare deserts, and transportation limitations can influence the risk of postoperative complications but have limited preexisting data. Proper nutrition has been found to aid in healing and can lessen postoperative complications.

Methods

We utilized the 2021 National Inpatient Sample from the Healthcare Cost and Utilization Project (HCUP) to analyze the association of food insecurity with common postoperative complications for hernia repair, cholecystectomy, gastric bypass, gastric banding, and enterectomy.

Results

Multivariate logistic regression adjusting for demographics (age, gender, and race) and comorbidities (diabetes, hypertension, cancer, substance misuse, autoimmune disorders, mental health, chronic obstructive pulmonary disorder (COPD), obesity, vascular disease, and thyroid disorders) showed a 4.5% increase in the risk of postoperative complications in patients at risk of having food insecurity (odds ratio (OR)=1.045; 95% confidence interval (CI): 1.004-1.087; p=0.03).

Conclusion

This significant increase in risk can be used as a predictor to further refine patient care related to postoperative complications. Providers can use diagnosis codes associated with socioeconomic risk factors in patient charts to flag the patients for additional precautions and follow-up. Additionally, there is a need for more healthcare providers and facilities in areas of food shortage.

## Introduction

The risk of postoperative complications varies dramatically from as low as 4% to as high as 43.5% [1,2]. These disparities highlight the complexity and individuality of postoperative recovery, with each patient’s risk profile influenced by a wide array of factors. Defined as any adverse or undesirable event following surgery, postoperative complications can range from mild discomfort, such as nausea and swelling, to life-threatening issues, such as infection and pneumonia [3]. The impact of surgery on the body is substantial, regardless of the procedure’s severity. Healing after surgery requires the body to generate new cells, a process that demands significantly more energy and nutrition than usual [4,5]. Adequate nutrition plays a crucial role in this recovery process, as malnutrition slows healing and leads to longer hospital stays, both causing increases in the risk of postoperative complications [4-6]. Additionally, patients already experiencing nutritional deficiencies before a procedure have impaired immune systems, making them more susceptible to infections and other perioperative complications [7]. The multifactorial causes of postoperative complications have proven difficult to predict and treat prophylactically. Much research has been done to prevent some of these symptoms; however, preventative measures are often overlooked by clinicians, especially for mild symptoms [8]. Despite extensive research into preventative measures, these complications remain challenging to predict.

Socioeconomic risk factors, such as food insecurity, are closely tied to malnutrition and play a crucial, although often overlooked, role in predicting surgical outcomes. Food security is defined as a person having consistent access to enough food to maintain an active and healthy life [9,10]. Food access can be limited due to a variety of social, financial, environmental, and cultural factors, causing someone to be deemed food insecure. In 2023, 13.5% of US households experienced food insecurity, with particularly high rates among households with children, single parents, Black and Hispanic communities, and those living in rural and southern areas [11]. Previous studies found that food-insecure patients located in areas with a higher-than-average median income had higher rates of mortality and developing an ileus, as well as longer hospital stays [12]. The multifactorial relationship between food security and postoperative outcomes encompasses socioeconomic factors, healthcare access, psychological stress, and inadequate nutritional knowledge, which may all converge to worsen the health of food-insecure individuals, especially in a surgical setting. As food insecurity has become an increasingly significant public health issue, it has garnered attention in academic research, but little has been done to mitigate its effects, especially in relation to surgical outcomes.

Malnutrition is a well-documented and significant contributor to poor surgical outcomes. Studies have shown that malnourished patients experience slower wound healing, increased rates of infection, prolonged hospital stays, and a higher risk of mortality [13]. This is particularly true in the context of abdominal surgery, where the body’s ability to heal postoperatively is heavily reliant on nutritional support [14]. The gastrointestinal and abdominal regions are particularly vulnerable to the effects of poor nutrition due to their role in digestion and absorption. Given the complexity and prevalence of abdominal surgeries, further exploration is needed to assess the impact of food insecurity on postoperative outcomes.

In this study, we aimed to quantify the incidence of postoperative complications (e.g., infection, hemorrhage, and sepsis) following abdominal surgeries such as hernia repair, cholecystectomy, gastric bypass, gastric banding, and enterectomy using the 2021 National Inpatient Sample (NIS) data. Further, we analyzed the association of socioeconomic risk factors of food insecurity, healthcare access, and transportation limitations with postoperative complications and hospital length of stay. We hypothesize that patients who experience food insecurity and reside in areas with limited access to healthcare are at an increased risk for postoperative complications and prolonged hospital stays following abdominal surgery, independent of their clinical comorbidities. By identifying these risk factors, we aim to underscore the importance of incorporating nutrition-based interventions into preoperative care to improve patient outcomes.

## Materials and methods

We included data on 6.6 million unweighted patients from the Healthcare Cost and Utilization Project’s (HCUP) National Inpatient Sample (NIS) 2021 data [15]. The database includes a 20% stratified sample of all discharges from US community hospitals, which are nonfederal, short-term, general, and specialty hospitals, excluding rehabilitation and long-term acute care hospitals. The database represents over 97% of the US population with data from 47 states and the District of Columbia. Sample charts from the HCUP database were obtained from each hospital and weighted based on patient volume.

After applying NIS weights, the study included 2,632,219 weighted cases of patients who had undergone a gastrointestinal surgery, including hernia repair, cholecystectomy, gastric bypass, laparoscopic gastric banding, or enterectomy, based on the Current Procedural Terminology (CPT) codes. The most common postoperative complications following the surgery were selected based on the frequency of cases (Appendices). Of the patients who had undergone gastrointestinal surgery, 382,399 cases were found to develop complications, and the other 2,249,820 had no complications reported. The mean age for all cases was 54.87 years, and 57.82 years for patients who developed postoperative complications. Of the patients with complications, 60% were female, compared with 62% among all cases combined. The cases involved a majority White population.

The incidence of a postoperative complication was calculated as a categorical response variable indicating whether or not the patient experienced any of the common complications. Tenth revision of the International Classification of Diseases (ICD) codes were obtained from the American Medical Association coding resources [16]. All ICD-10 codes for complications were used to be specific to postoperative complications due to a procedure. Food insecurity was defined as a patient living in an area with an urban-rural classification of 6 (the most rural) and in a ZIP code with an average income level in the first quartile [17]. The urban-rural classification was intended by NCHS to quantify health risk. This variable was further strengthened by including the income quartile provided by the database. Logistic regression analysis was conducted to measure the association of food insecurity and other sociodemographic variables with the patient experiencing a postoperative complication among patients with common gastrointestinal surgeries. We also utilized a generalized gamma log-linear model to assess the association of food insecurity with a patient's length of hospital stay following the surgery. We adjusted both the logistic regression and generalized gamma log-linear analyses for the sociodemographic variables of age, sex, race/ethnicity (White, African American, Native American, Asian, Hispanic, and other), hypertension with and without complications, diabetes mellitus (DM) with and without complications, cancer, acquired immune deficiency syndrome (AIDS), alcohol use disorder, autoimmune disorders, depression, dementia, drug use disorder, chronic obstructive pulmonary disorder (COPD), obesity, peripheral vascular disease (PVD), hyperthyroidism and other thyroid disorders, and hypothyroidism. Statistical significance was defined as p<0.05. We used SPSS 31 software (IBM Corp., Armonk, NY) for all the analyses.

## Results

**Figure 1:**
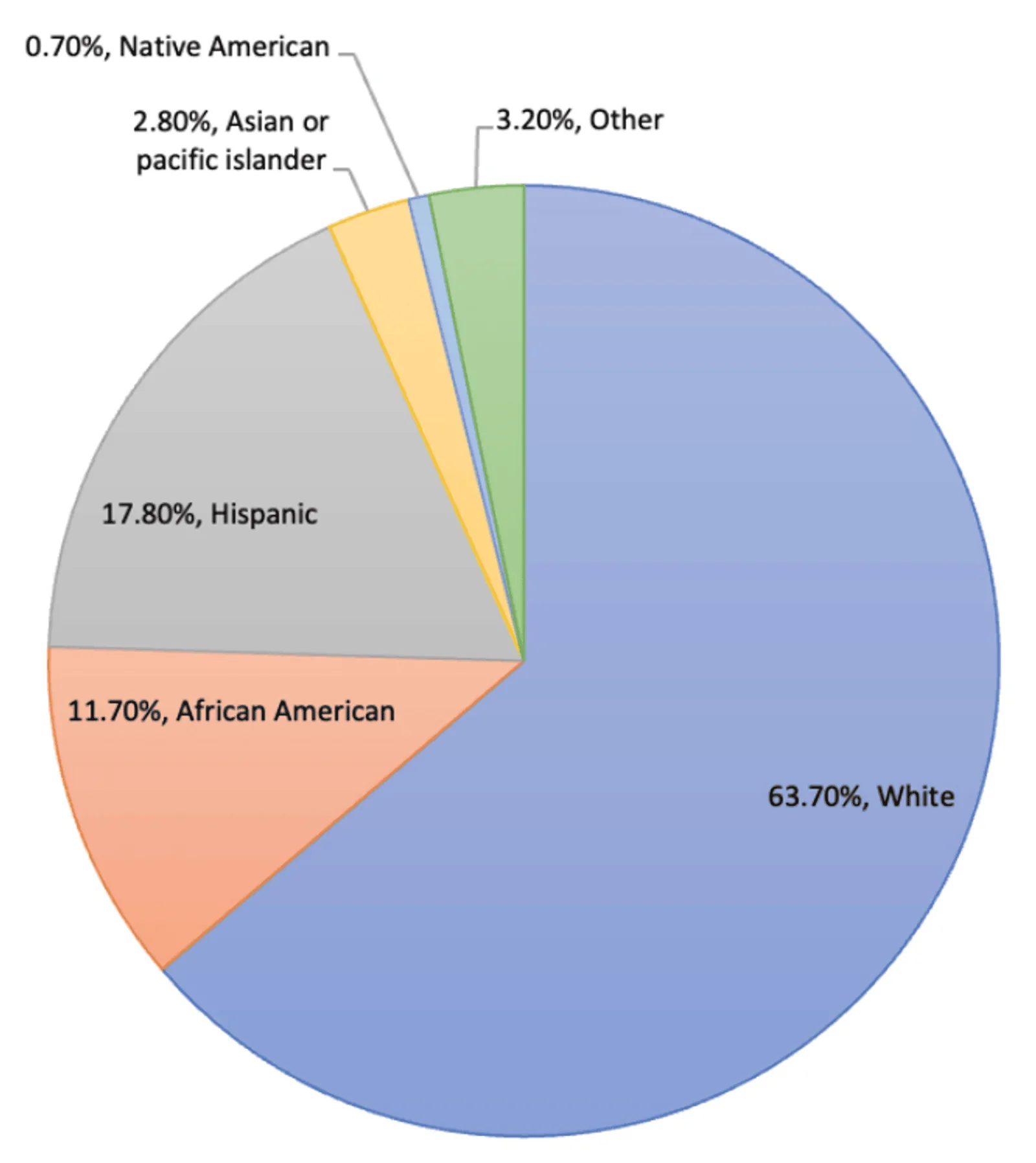
Racial demographics of the weighted National Inpatient Sample population undergoing abdominal surgeries, displayed as a percentage of total patients

Of the 2,632,219 patients who had undergone gastrointestinal surgery, 382,399 cases were found to develop complications, and the other 2,249,820 had no complications reported (Table 1). Additionally, 98,149 patients were deemed food insecure according to the variable definition, 15,350 (15.64%) of whom experienced complications (Table 1). A total of 2,167,021 patients were deemed food secure, with 367,049 (14.48%) of these patients experiencing a post-surgical complication (Table 1). The racial demographic data of the cohort studied are provided in Figure 1. 

**Table 1:** Raw weighted values for postoperative complications in food-secure and food-insecure patient groups

	Complications	No complications	Total
Food insecure	15,350	82,799	98,149
Food secure	367,049	2,167,021	2,534,070
Total	382,399	2,249,820	2,632,219

The unadjusted model demonstrated a significant association between food insecurity and postoperative complications (odds ratio (OR)=1.095; 95% confidence interval (CI): 1.052-1.138; p<0.001) (Table 2). After inclusion of demographic variables and comorbidities, the adjusted analysis continued to show a statistically significant association (adjusted OR=1.045; 95% CI: 1.005-1.087; p=0.029) (Table 3). Cancer was among the strongest predictors, with affected patients experiencing a 66.5% increase in the odds of complications (OR=1.665; 95% CI: 1.627-1.704; p<0.001). Other conditions associated with increased complication risk included hyperthyroidism (OR=1.471; 95% CI: 1.382-1.566), AIDS (OR=1.519; 95% CI: 1.357-1.701), autoimmune disease (OR=1.364; 95% CI: 1.306-1.424), chronic obstructive pulmonary disease (OR=1.216; 95% CI: 1.191-1.241), peripheral vascular disease (OR=1.190; 95% CI: 1.146-1.235), depression (OR=1.208; 95% CI: 1.181-1.236), drug abuse (OR=1.198; 95% CI: 1.135-1.265), obesity (OR=1.127; 95% CI: 1.109-1.148), and alcohol abuse (OR=1.134; 95% CI: 1.083-1.188) (all p<0.001). Increasing age was associated with a small but consistent increase in complication risk, with each additional year associated with a 0.9% increase in odds (OR=1.009; 95% CI: 1.008-1.009; p<0.001).

**Table 2:** Logistic regression results assessing food insecurity’s bivariate association with postoperative complication risk

Risk factor	Regression coefficient (B)	p-value	Odds ratio	95% confidence interval
Food insecurity	0.090	<0.001	1.095	1.052-1.138

**Table 3:** Multivariable generalized linear model assessing the association between demographic characteristics and comorbidities and hospital length of stay Regression coefficients represent adjusted differences in hospital length of stay (days) associated with each risk factor. All associations shown were statistically significant (p<0.001) except for non-White race. AIDS: acquired immune deficiency syndrome, COPD: chronic obstructive pulmonary disorder

Risk factor	Regression coefficient (B)	Standard error	95% confidence interval
Food insecurity	0.097	0.0069	0.083-0.111
Minor diabetes mellitus	1.104	0.0041	1.096-1.112
Major diabetes mellitus	1.455	0.0059	1.443-1.466
Minor hypertension	0.648	0.0044	0.639-0.656
Major hypertension	0.785	0.0042	0.777-0.794
Non-White race	-0.001	0.0027	-0.007-0.001
Cancer	1.148	0.0055	1.137-1.159
Age	0.014	0.00007	0.014-0.015
Alcohol abuse	0.795	0.0064	0.782-0.808
AIDS	0.710	0.0185	0.674-0.746
COPD	0.179	0.0037	0.172-0.186
Peripheral vascular disease	0.617	0.0062	0.605-0.629
Hypothyroidism	0.027	0.0045	0.018-0.035
Hyperthyroidism	0.317	0.0126	0.292-0.341
Obesity	0.572	0.0037	0.565-0.579
Dementia	1.187	0.0063	1.175- 1.200
Drug abuse	1.054	0.0064	1.041-1.066
Female	-0.734	0.0028	-0.739-(-0.728)
Autoimmune	0.398	0.0083	0.382-0.414
Depression	0.268	0.0044	0.260-0.277

**Figure 2:**
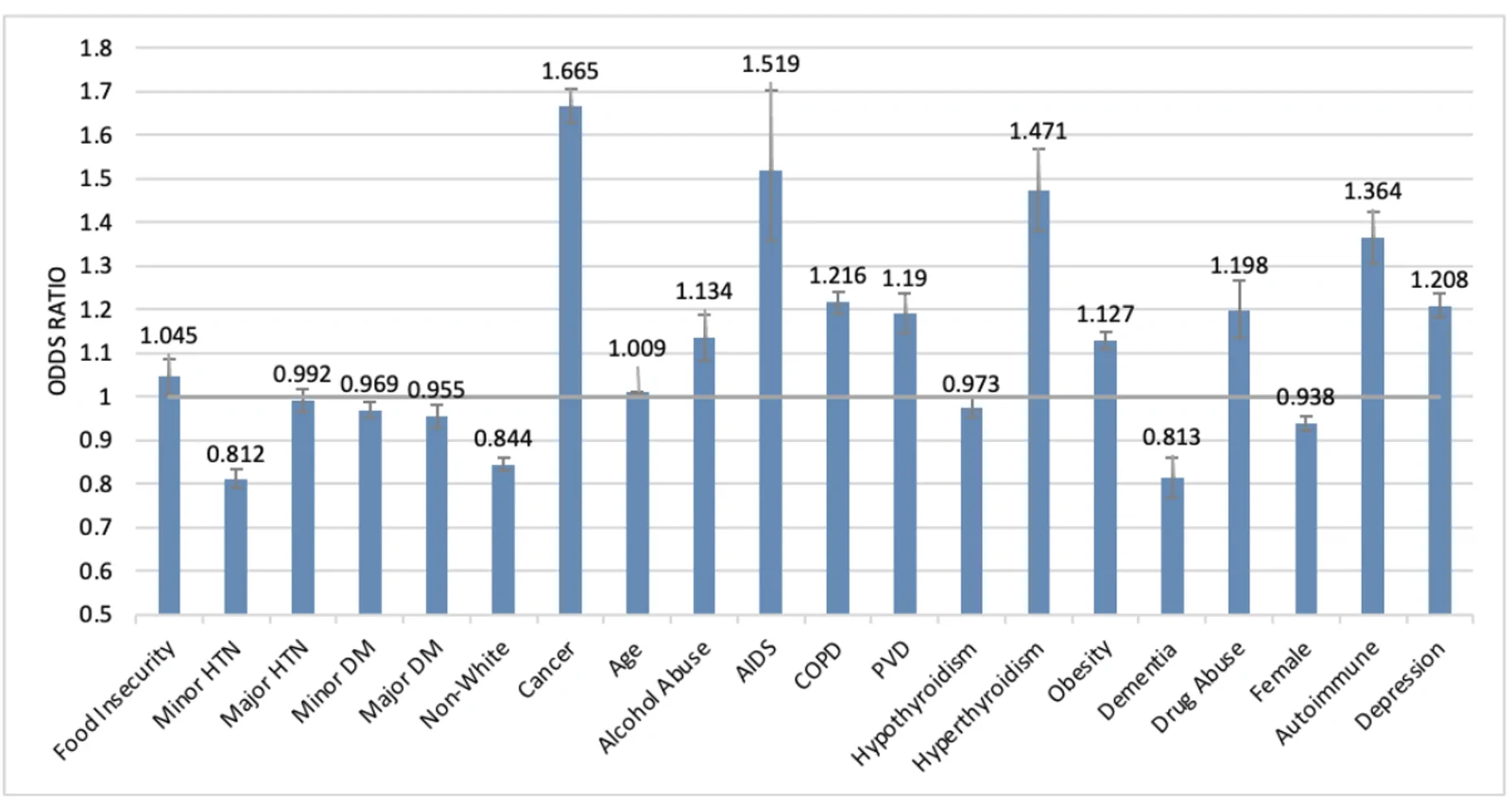
Odds ratios for postoperative complications by demographic characteristics and comorbidities Bars represent odds ratios from the multivariable logistic regression model, and error bars indicate 95% confidence intervals. Minor hypertension and diabetes mellitus indicate diagnoses without systemic complications, whereas major hypertension and diabetes mellitus indicate diagnoses with systemic complications. Non-White race includes Native American, Asian, African American, Hispanic, and other. Error bars represent 95% confidence intervals. The horizontal reference line denotes an odds ratio of 1.0. DM: diabetes mellitus, HTN: hypertension, AIDS: acquired immune deficiency syndrome, COPD: chronic obstructive pulmonary disorder, PVD: peripheral vascular disease

Several factors were associated with lower odds of postoperative complications. Minor diabetes mellitus was associated with an 18.8% reduction in odds (OR=0.812; 95% CI: 0.791-0.833; p<0.001), whereas major diabetes mellitus was not significantly associated with complications (OR=0.992; 95% CI: 0.966-1.018; p=0.523). Both minor hypertension (OR=0.969; 95% CI: 0.951-0.987) and major hypertension (OR=0.955; 95% CI: 0.929-0.982) were associated with slightly reduced odds of complications (p<0.001). Minor hypertension and diabetes mellitus are characterized as diagnoses without systemic complications, whereas major hypertension and diabetes mellitus represent diagnoses with systemic complications. Non-White race (OR=0.844; 95% CI: 0.829-0.859), dementia (OR=0.813; 95% CI: 0.768-0.861), and female sex (OR=0.938; 95% CI: 0.923-0.954) were also associated with lower complication risk. Hypothyroidism showed a small but statistically significant association with reduced odds of complications (OR=0.973; 95% CI: 0.950-0.997; p=0.027). Full regression results are presented in Table 3 and Figure 2.

Hospital stay length

In addition to overall complication rates, multiple social, demographic, and clinical risk factors were independently associated with length of hospital stay. All variables except non-White race were statistically significant (p<0.001) and demonstrated very small standard errors (SE) with narrow confidence intervals, reflecting the high precision of estimates afforded by the large sample size.

In an initial analysis examining food insecurity independently, food insecurity was associated with a significant increase in length of hospital stay (B=0.178, SE=0.0070; 95% CI: 0.164-0.192; p<0.001) (Table 4). After adjustment for demographic characteristics and medical comorbidities, the association between food insecurity and length of stay was attenuated but remained statistically significant (adjusted B=0.097, SE=0.0069; 95% CI: 0.083-0.111; p<0.001) (Table 5). Substantially larger increases in length of stay were observed for metabolic and cardiovascular conditions, including minor diabetes mellitus (B=1.104, SE=0.0041; 95% CI: 1.096-1.112), major diabetes mellitus (B=1.455, SE=0.0059; 95% CI: 1.443-1.466), minor hypertension (B=0.648, SE=0.0044; 95% CI: 0.639-0.656), and major hypertension (B=0.785, SE=0.0042; 95% CI: 0.777-0.794).

**Table 4:** Generalized linear model assessing the independent effect of food insecurity on hospital length of stay

Risk factor	Regression coefficient (B)	p-value	Standard error	95% confidence interval
Food insecurity	0.178	<0.001	0.0070	0.164-0.192

**Table 5:** Multivariable logistic regression analysis of demographic characteristics and comorbidities associated with postoperative complications Positive regression coefficients indicate increased odds of postoperative complications, whereas negative coefficients indicate decreased odds. Minor hypertension and diabetes mellitus represent diagnoses without systemic complications; major hypertension and diabetes mellitus represent diagnoses with systemic complications. AIDS: acquired immune deficiency syndrome, COPD: chronic obstructive pulmonary disorder

Risk factor	Regression coefficient (B)	p-value	Odds ratio	95% confidence interval
Food insecurity	0.044	0.029	1.045	1.005-1.087
Minor diabetes mellitus	-0.209	<0.001	0.812	0.791-0.833
Major diabetes mellitus	-0.008	0.523	0.992	0.966-1.018
Minor hypertension	-0.032	<0.001	0.969	0.951-0.987
Major hypertension	-0.046	<0.001	0.955	0.929-0.982
Non-White race	-0.170	<0.001	0.844	0.829-0.859
Cancer	0.510	<0.001	1.665	1.627-1.704
Age	0.009	<0.001	1.009	1.008-1.009
Alcohol abuse	0.126	<0.001	1.134	1.083-1.188
AIDS	0.417	<0.001	1.519	1.357-1.701
COPD	0.200	<0.001	1.216	1.191-1.241
Peripheral vascular disease	0.174	<0.001	1.190	1.146-1.235
Hypothyroidism	-0.027	0.027	0.973	0.950-0.997
Hyperthyroidism	0.386	<0.001	1.471	1.382-1.566
Obesity	0.121	<0.001	1.127	1.109-1.148
Dementia	-0.207	<0.001	0.813	0.768-0.861
Drug abuse	0.181	<0.001	1.198	1.135-1.265
Female	-0.064	<0.001	0.938	0.923-0.954
Autoimmune	0.310	<0.001	1.364	1.306-1.424
Depression	0.189	<0.001	1.208	1.181-1.236

Several chronic and severe medical conditions were among the strongest predictors of prolonged hospitalization. Cancer was associated with an increase of more than one day in length of stay (B=1.148, SE=0.0055; 95% CI: 1.137-1.159), as were dementia (B=1.187, SE=0.0063; 95% CI: 1.175-1.200) and drug abuse (B=1.054, SE=0.0064; 95% CI: 1.041-1.066). Alcohol abuse (B=0.795, SE=0.0064; 95% CI: 0.782-0.808) and AIDS (B=0.710, SE=0.0185; 95% CI: 0.674-0.746) were also associated with increases in length of stay.

Additional comorbidities were associated with smaller but consistent increases in hospitalization duration, including chronic obstructive pulmonary disease (B=0.179, SE=0.0037; 95% CI: 0.172-0.186), peripheral vascular disease (B=0.617, SE=0.0062; 95% CI: 0.605-0.629), obesity (B=0.572, SE=0.0037; 95% CI: 0.565-0.579), autoimmune disease (B=0.398, SE=0.0083; 95% CI: 0.382-0.414), depression (B=0.268, SE=0.0044; 95% CI: 0.260-0.277), hyperthyroidism (B=0.317, SE=0.0126; 95% CI: 0.292-0.341), and hypothyroidism (B=0.027, SE=0.0045; 95% CI: 0.018-0.035). Age was associated with a small but consistent increase in length of stay (SE=0.00007; 95% CI: 0.014-0.015). Female sex was associated with a shorter length of stay compared with male sex (B=-0.734, SE=0.0028; 95% CI: -0.739 to -0.728). Non-White race was not significantly associated with hospital length of stay (B=-0.001 days; 95% CI: -0.007 to 0.001), indicating no meaningful difference in adjusted length of stay by race. Full regression results for length of stay, including all covariates, are presented in Table 5.

## Discussion

The role of food insecurity in postoperative outcomes is magnified when considering the multifaceted ways in which it affects patients. Nutritional deficiencies associated with food insecurity can lead to a weakened immune system, increasing the risk of infection following surgery [7]. Patients undergoing abdominal surgeries in particular may face compounded risks, making food insecurity a key but often overlooked factor in predicting operation outcomes [18]. Our study found that individuals experiencing food insecurity had a 9.5% higher incidence of complications compared to food-secure patients (Table 2). Even when comorbidities and demographic factors were accounted for, food insecurity still predicted a 4.5% higher incidence of complications (Table 3, Figure 2). Although food insecurity showed a smaller effect size on complication rates when controlling for other variables, it remained a statistically significant predictor. Clinically, the associated 4.5% increase in complication rates represents a substantial and potentially preventable risk factor that warrants attention during the preoperative evaluation. Several factors were associated with lower odds of postoperative complications, including minor diabetes mellitus, hypertension, dementia, female sex, and non-White race. These findings should not be interpreted as evidence of a protective biological effect; rather, they likely can be attributed to a combination of surgical selection bias, differential perioperative management, and variation in complication recognition and coding. Patients with known chronic conditions who undergo elective surgery are often carefully selected and more closely monitored, potentially reducing the likelihood of reported complications. Additionally, competing risks may further contribute to these observed associations.

Food insecurity was also associated with slightly, but still significantly, longer hospital stays when compared to food-secure individuals (Table 4). This effect persisted even after adjusting for other comorbidities and risk factors, although the magnitude was relatively smaller (Table 5). While chronic conditions such as diabetes, hypertension, and cancer demonstrated a stronger individual association with postoperative length of stay, food insecurity still emerged as a significant independent predictor contributing to both prolonged hospitalization and increased complication rates. Previous research has shown that food insecurity has a substantial impact on the health of patients with chronic illnesses [19], which may help explain why its effect on length of stay appears attenuated despite a more pronounced association with complication rates. Although food insecurity was independently associated with increased hospital length of stay, non-White race was not significantly associated with length of hospitalization after adjustment for comorbidities and demographic factors. This suggests that differences in length of stay are more strongly driven by socioeconomic and clinical factors rather than race itself. These findings highlight the importance of addressing food insecurity as an independent risk factor for prolonged postoperative recovery.

A key challenge is that food insecurity is not routinely addressed in the preoperative phase for elective procedures [18]. Nutrition-based interventions, such as preoperative nutritional supplementation or referrals to dietitians, are frequently not prioritized, particularly when dealing with mild symptoms or issues that do not seem immediately life-threatening [20,21]. While immediate preoperative fasting is still recommended to prevent aspiration, proper nourishment for the surgery requires long-term preparation [22]. Unfortunately, this may stem from the perception that mild nutritional deficits will not significantly impact the healing process. Although patients may be consuming enough calories, providers should focus on the quality of their intake, not just the quantity. Assessment of food insecurity and other socioeconomic risk factors remains minimal across medical specialties as a whole [23]. However, the chronic nature of food insecurity, coupled with its negative impact on immune function and wound healing, suggests that a more proactive and integrated approach is necessary [24]. The 9.5% increased odds of complications in food-insecure individuals highlight the need for greater attention to this issue, particularly to improve outcomes after abdominal surgery. Elective procedures provide a unique opportunity for intervention, as there is time to address and prepare for nutritional deficits before surgery. In contrast, urgent and emergent surgeries may not allow for preoperative nutritional preparation, increasing the likelihood that food insecurity goes unaddressed in these settings.

Given the increasing recognition of social determinants of health, healthcare systems should consider policies that account for food insecurity as a key social determinant of postoperative outcomes. Clinicians should be trained to recognize the signs of food insecurity, and multidisciplinary care teams could collaborate to offer holistic care that addresses the patient’s nutritional, social, and psychological needs. Importantly, food security screening should be seen as part of a broader effort to reduce health inequities and improve patient care in vulnerable populations. Future studies are suggested to focus on quantifying the effects of food insecurity on both the complication rates and the severity of complications. In addition to this, better identifying methods that address the barriers contributing to food insecurity in the preoperative stage could help lower its postoperative effects. Longitudinal studies could also be beneficial in tracking differences in recovery in patients who are not experiencing food insecurity compared to those who are. Lastly, programs designed to increase the assessment of patients’ socioeconomic risk factors should continue to be researched to express the need for implementing clinical guidelines.

Certain limitations should be considered when interpreting these findings. HCUP data is primarily an administrative database collected from coding for billing and reimbursements, making it an inexact fit for measuring complications and severity. Laboratory values, imaging results, and other pertinent information could not be measured in this study but should be a larger focus in future research. We used a zip code-related proxy variable to measure food insecurity, which required assumptions based on location and median household income. Using an individual-level measurement of income and access to food sources would allow for more precision and be a better indicator of food security. Finally, the NIS dataset only looks at inpatient data, restricting the ability to make generalized conclusions for all clinical settings and non-hospitalized individuals. 

## Conclusions

Food insecurity significantly increased both the incidence of complications and the length of stay in patients undergoing abdominal surgery. Patients experienced a 9.5% increased risk of complications when considering food insecurity independently and a 4.5% increased risk when including demographics and comorbidities. Food insecurity had a less robust, but still significant, effect on length of hospital stay. To help lessen postoperative complications, providers should acknowledge the disparities of patients living in rural areas and the effect it will have on their healthcare journey. By doing this, clinicians should use the ICD-10 codes associated with socioeconomic risk factors to identify patients for additional precautions and follow-up. Additionally, patients should be educated on proper nutrition prior to elective surgeries. If the patient requires emergency or urgent surgery, the clinician should properly assess their nutritional status and prepare for the patient needing additional support postoperatively. While postoperative complications will always be an inherent risk of surgery, food insecurity represents a modifiable factor that, if addressed proactively, could significantly reduce the incidence and severity of these complications. This requires a shift in how we approach preoperative care to integrate nutritional screening, targeted interventions, and awareness of the broader socioeconomic and environmental factors impacting health. Ultimately, addressing food insecurity and ensuring access to adequate nutrition could reduce postoperative complications, shorten hospital stays, and support patients’ recovery.

## Appendices

Postoperative complications

The following are the most common postoperative complications following surgery: peritonitis, anastomotic leak, retroperitoneal abscess, urinary tract infection, cystitis, pyelonephritis, colostomy site obstruction/prolapse or other complication, fistula, diverticular disease of the colon without perforation, diverticulitis of the colon with abscess, mechanical complication of a colostomy, mechanical complication of graft, perforation of the intestine, short bowel syndrome, vitamin/mineral deficiencies due to non-dietary reasons, calculus of the gallbladder with acute cholecystitis, post-gastric surgery syndrome/dumping syndrome, infection after gastric band procedure, infection after bariatric surgery, other complications of bariatric surgery, other complications of gastric banding, vomiting after gastrointestinal surgery, post-surgical malabsorption, postprocedural obstruction, gastrojejunal ulcer, duodenal ulcer, graft rejection, mechanical complication of a gastric band, complications of an implanted device, other specified diseases of the stomach, gastroparesis, infection following a procedure, peritoneal abscess, dehiscence of surgical wound, post-cholecystectomy syndrome, post-surgical bleeding of digestive system structures, bile leak, injury to biliary tract, other cholelithiasis with complications, cholangitis, recurrent hernia after surgical procedure, urinary retention, infection and inflammatory reaction due to a prosthetic implant or graft, seroma, hematoma, postprocedural seroma of a digestive system organ, paralytic ileus, postprocedural shock, acute embolism and thrombosis, pulmonary embolism, anesthesia complications, shock due to anesthesia, other complications of anesthesia, malignant hyperthermia, hypertrophic scar, keloid scar, scar tissue, other respiratory disorders due to surgery, pneumonia, stoma sequelae of surgical procedures, acute pain, chronic pain, complication of mesenteric artery following a procedure, postoperative fever, and sepsis.
